# Predictors of mortality in hemodialysis patients

**DOI:** 10.11604/pamj.2019.33.61.18083

**Published:** 2019-05-28

**Authors:** Rajaa Msaad, Rajaa Essadik, Karima Mohtadi, Hasnaa Meftah, Halima Lebrazi, Hassan Taki, Anass Kettani, Ghizlane Madkouri, Benyounes Ramdani, Rachid Saïle

**Affiliations:** 1 Laboratory of Biology and Health, URAC 34, Hassan II University-Casablanca, Faculty of Sciences Ben M’Sik, Avenue Cdt Driss El Harti BP 7955, Sidi Othmane, Casablanca, Morocco; 2 Department of Nephrology-Transplantation and Hemodialysis of the University Hospital Center Ibn Rochd of Casablanca, Casablanca, Morocco

**Keywords:** Mortality, under nutrition, cardiovascular diseases, and chronic renal failure

## Abstract

**Introduction:**

Mortality in patients with chronic renal failure is high compared to the general population. The objective of our study is to evaluate the predictive factors related to mortality in hemodialysis.

**Methods:**

This is a retrospective study involving 126 hemodialysis patients in the Nephrology Department of Ibn Rochd Hospital, Casablanca. Data were collected between January 2012 and January 2016. For each of our patients, we analyzed demographic, clinical, biological and anthropometric data. The Kaplan-Meier method and the log-rank test were used to evaluate and compare survival curves. To evaluate the effect of predictors of mortality, we used the proportional Cox hazard model.

**Results:**

The analysis of the results showed that the surviving patients were younger than the deceased patients (43.07±13.52 years versus 53.09±13.56 years, p=0.001). Also, the latter has a significantly lower albumin and prealbumin levels (p=0.01 and p=0.04 respectively). Overall survival was 80.2%. Cox regression analysis at age (HR=1.26, p<0.0002), inflammation (HR=1.15, p<0.03), AIP> 0.24 (HR=2.1, p<0.002) and cardiovascular disease (RR=2.91, p<0.001) were associated with global and cardiovascular mortality.

**Conclusion:**

Our study showed that the mortality rate is high in our cohort. In addition, cardiovascular diseases, under nutrition and inflammation are predictive factors for mortality. Treatment and early management of these factors are essential for reducing morbidity and mortality.

## Introduction

Over the last two decades, advances in the treatment of chronic kidney disease (CKD) helped increase the survival of hemodialysis (HD) patients. There are currently about 2 million people on hemodialysis worldwide, representing only 10% of people who need it [1]. This number continues to grow with the aging of the population. Unfortunately, despite the technical advances in hemodialysis, the mortality of patients with end-stage renal disease (ESRD) is 10 to 30 times higher than that of the general population [2, 3]. In 2011 data from the Maghreb showed that the mortality rate among patients varied between countries ranging from 6% in Morocco, and 10.4% in Tunisia to 12% in Algeria [4]. This mortality is partly explained by the presence of important co-morbidities, such as cardiovascular diseases, diabetes and advanced age. At the same time, among the other risk factors for mortality, under nutrition is a proven factor, widely studied but too often forgotten and underestimated [5]. Protein-energy malnutrition in dialysis patient is frequent and multifactorial which begins well before the start of hemodialysis. It results from an imbalance between the contributions and the needs of the organism, resulting in tissue losses having deleterious functional consequences, associated with a high morbidity and with an unfavorable prognosis, as was confirmed in our previous studies [6, 7]. In fact, the annual mortality of undernourished HD patients is close to 30%, whereas it is generally 10 to 15% in non-malnourished patients [8]. Note that in this population, overweight and obesity paradoxically appear as factors of good prognosis [9]. However, the mechanisms involved in the high mortality in the context of under nutrition are still poorly understood [10]. In this context, we conducted a retrospective study to determine the factors implicated in mortality in long-term hemodialysis patients. We also tried to evaluate the impact of protein-energy malnutrition on the risk of mortality in order to verify whether the cause of death of patients followed for an ESRD was renal or rather caused by other pathologies. For this, we examined the nutritional, lipid and inflammatory profiles of our study population.

## Methods

This is a descriptive retrospective study, which took place over a period of 4 years from January 2012 to January 2016 in the Department of Nephrology-Transplantation and Hemodialysis of the University Hospital Centre of Casablanca, Morocco. In 2012, we included 126 hemodialysis patients (66 women and 60 men) in our study. During this period 22 patients died. Patients with chronic dialysis for less than 6 months, patients with cancer at the time of inclusion, pregnant women, amputee patients or patients with a pacemaker, since this makes impedance measurement impossible, have been excluded. The causes of death were identified by differentiating deaths directly related to CKD, from those caused by cardiovascular causes or other causes (protein-energy malnutrition, high blood pressure, etc.).

For each of our patients, we analyzed the demographic, clinical, biological and anthropometric data. Clinical data were collected from patients records which include: sex, age, hemodialysis duration in months, initial nephropathy, and blood pressure. We also chosed to study as biological parameters: albumin, prebuminemia, serum calcium, phosphatemia, lipid profile (total cholesterol, HDL-cholesterol, LDL-cholesterol, triglycerides), lipid ratios including the atherogenic index plasma (AIP), hemoglobin and C-reactive protein (CRP). In this study we defined the presence of under nutrition based on serum albumin (S.Albumin): presence of under nutrition if S.Albumin <38 g/L and no under nutrition if S.Albumin ≥38g/L.

**Statistical analyses:** we analyzed the data using SPSS software version 21.0. First, we performed a descriptive analysis of the sample as a whole; the quantitative variables were expressed as mean ± standard deviation, and the qualitative variables as frequencies and percentages. The ANOVA test was chosen for the comparison of quantitative variables and the Chi 2 or Fisher exact test for the comparison of qualitative variables. The distribution of the delay in the occurrence of death was first described by the Kaplan-Meier estimator. All variables are studied in univariate (log-rank). To evaluate the effect of the different predictors of mortality for any cause, we used the proportional hazard Cox model to include the parameters most significantly associated with survival. The association between each of the explanatory variables and the time to death was tested with a univariate Cox model. The variables were then included in the multivariate Cox model if their p-value was less than 0.20 in univariate analysis. For all analyses, the significance threshold was set at 0.05.

## Results

Presentation of the study population: in the present study, the mean age of participants was 44.81±14.01 years old. Of 126 subjects, 52.38% were females and 47.62% were males. We compared patients according to their “surviving” or “deceased” status. The demographic and clinical data of the two groups are listed in Table 1. The analysis of the results obtained showed that the surviving patients were younger than the deceased patients (43.07±13.52 years versus 53.09±13.56 years old, p=0.001). In addition, these patients have a significantly lower albumin and prealbumin levels compared to the surviving patients (p=0.01 and p=0.04 respectively). The comparison of lipid and lipoprotein parameters did not show any significant differences between the two groups. In contrast, the inflammatory marker (CRP) was significantly elevated in the deceased patients (p=0.006). Table 2 shows the biological and nutritional characteristics of the deceased patients. This description allows us to show the importance of the comorbidity of patients. Based on the main criterion used to characterize undernutrition (albumin <38 g/l), our results show that 77.27% of the dead patients were malnourished, 81.81% had anemia, 63.63% suffered from hypercholesterolemia, and 13.63% were diabetic. Several potential causes of death existed in the same patient. Cardiovascular events were the leading cause of death in our patients (22.72%), followed by stroke (13.64%) and infectious diseases (9.10%). We also noted 12 fatalities with undetermined causes (54.54%).

**Table 1:** Clinical and biological characteristics of the study population

Variables	Decades (n=22)	Survivals (n=94)	P value
Age (year)	53.09±13.56	43.07±13.52	**0.001**
Gender (F/H)	12/10	54/40	0.254
Duration of hemodialysis (months)	150.05±75.40	143.00±73.30	0.63
Blood pressure (mmHg)	130.00±24.30	126.08±16.84	0.182
Albumin (g/l)	35.37±3.80	37.92±5.36	**0.01**
Prealbumin(mg/l)	241.64±83.97	276.50±93.90	**0.04**
CRP (mg/l)	42.93±41.25	24.76±30.21	**0.006**
Hemoglobine (g/dl)	9.40±1.04	9.66±1.58	0.44
Calcemia (mg/l)	86.40±12.20	87.72±10.21	0.63
Phosphoremia (mg/l)	39.77±15.67	43.11±17.61	0.41
PTH (pg/ml)	574.18±585.05	486.40±577.98	0.58
Weight(kg)	60.02±12.50	57.60±12.57	0.43
BMI (kg/m^²^)	22.60±4.02	23.10±5.50	0.340
CT (g/l)	1.43±0.32	1.53±0.37	0.127
LDL-C (g/l)	0.65±0.35	0.78±0.36	0.06
HDL-C (g/l)	0.44±0.19	0.54±0.31	0.09
TG (g/l)	1.37±0.65	1.21±0.47	0.14
Non-HDL-C	2.55±0.74	2.64±1.16	0.35
TC/HDL-C	3.90±2.74	3.57±1.24	0.26
LDL-C/HDL-C	2.22±2.31	1.65±1.03	0.13
TG/HDL-C	3.30±3.05	2.91±1.41	0.27
Non-HDL/HDL-C	2.90±2.74	2.57±1.24	0.30
AIP	0.42±0.18	0.38±0.34	0.32

**BMI:** Body mass index; **CRP:** *C-reactive protein;* **TC:** Total cholesterol; **TG:** Triglyceride; **HDL-C**: High-Density Lipoprotein-Cholesterol; **Non-HDL-C:** Non-high density lipoprotein-cholesterol; **LDL-C:** Low-Density Lipoprotein-Cholesterol; **AIP=** Atherogenic Index of Plasma.

**Table 2:** Prevalence of comorbidities in deceased hemodialysis patients (n= 22)

Variables	n (%)
Age > 60 years	12(54.54)
Albumin < 38 g/L	17(77.27)
Prealbumin < 300 mg/L	18(81.81)
Total cholesterol > 1.5 g/L	14(63. 63)
Fasting glucose >1.26 g/L	3(13.63)
Hemoglobin < 10 g/L	18(81.81)
CRP > 10 mg/L	16(72.72)
BMI< 23kg/m^²^	14(63.63)

**Figure 1:**
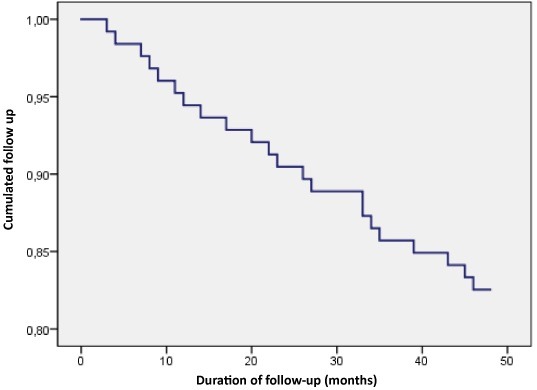
Patient overall survival curve during follow-up period

**CRP:** *C-reactive protein;* **BMI:** Body mass index;

Predictors of mortality in hemodialysis patients: Cox regression analysis demonstrated that age, inflammation, elevated AIP (>0.24) and the presence of cardiovascular diseases are associated with global and cardiovascular mortality independently (univariate analysis) and when they are associated (multivariate analysis). Moreover, analysis of Kaplan-Meier survival over a four-year period revealed that advanced age, increased CRP and AIP are associated with decreased survival. The multivariate Cox model was performed on a sample of 116 patients. The proportionality of risks in this model was verified for each covariate, with adjustment for other factors (age, sex). Multivariate analysis identified five potential risk factors (Table 3). Among these factors, we note that a patient aged over 65 is 1.26 times more likely at risk than a patient under 65 years of age (p=0.002). Also, having at least one cardiovascular pathology constitutes a risk of death in the HD patient 2.9 times greater compared to a patient without a cardiovascular pathology (p=0.001). It is also noted that malnourished patients have a relative risk of dying almost twice greater than non-malnourished patients (p=0.01). Thus, the survival analysis showed that the average survival since inclusion of our patients is 43.67 months. In addition, the overall survival at 12 months of follow-up was approximately 95%, 87.3% at 24 months and it reached 80.2% at 48 months as shown in Figure 1.

**Table 3:** Predictors of mortality according to the proportional hazard Cox model

Variables	HR	IC 95%	P value
Gender *(H/F)*	0.88	0.38-2.04	0.772
Age *(>60 years)*	1.26	1.11-3.074	**0.002**
Duration of hemodialysis (months) *(>10)*	0.81	0.68-1.95	0.563
Cardiovascular diseases	2.91	1.3-7.0	**0.001**
Undernutrition *(Albumine<38g/l)*	1.85	1.35-2.90	**0.01**
Diabetes (*blood glucose> 1.26 g/ l)*	0.67	0.2-3.7	0.75
Inflammation *(CRP>10mg/l)*	1.15	1.01-1.25	**0.03**
AIP *(>0.24)*	2.1	1.30-5.64	**0.002**
BMI *(<23 kg/m^²^)*	1.37	0.57-3.27	0.474
Serum prealbumin(*<300mg/l)*	0.62	0.21-1.83	0.38

**AIP=** Atherogenic Index of Plasma; **BMI:** Body mass index

**Figure 2:**
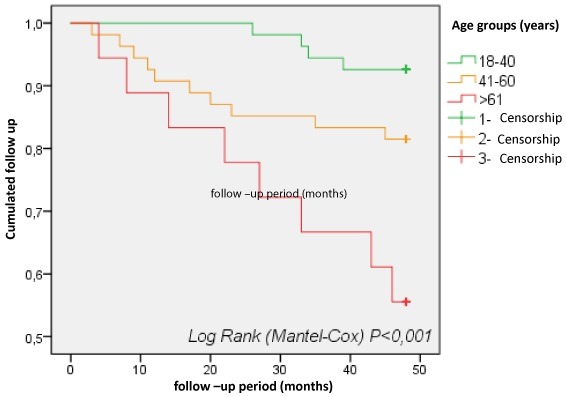
Survival curves of hemodialysis patients according to age groups

Variable age of patients: the survival of HD patients obviously depends on age (Figure 2). It is 82% after 4 years of follow-up in patients under 60, against 53% in those over 61 years.

**Figure 3:**
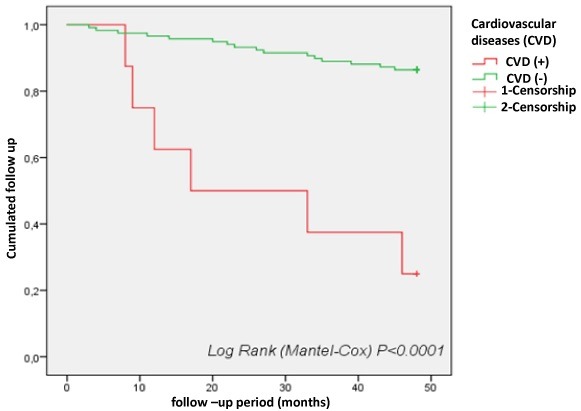
Survival curves for cardiovascular diseases

Cardiovascular diseases (CVD): the analysis of the survival curve showed that the presence of cardiovascular diseases is a factor favoring death in our population (p=0.0001). Survival at 48 months was 22% for patients with CVD and less than 90% for patients without CVD (Figure 3).

**Figure 4:**
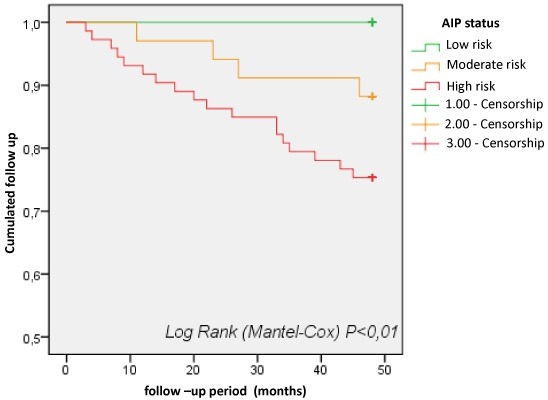
Survival curves of hemodialysis patients according to their AIP

Atherogenic Index of Plasma (AIP): from Figure 4, it can be seen that a high rate of AIP is a contributing factor to death in our population (p=0.01). That said, survival up to 48 months was 76% for patients with high cardiovascular risk, while it was 100% for patients with low risk.

**Figure 5:**
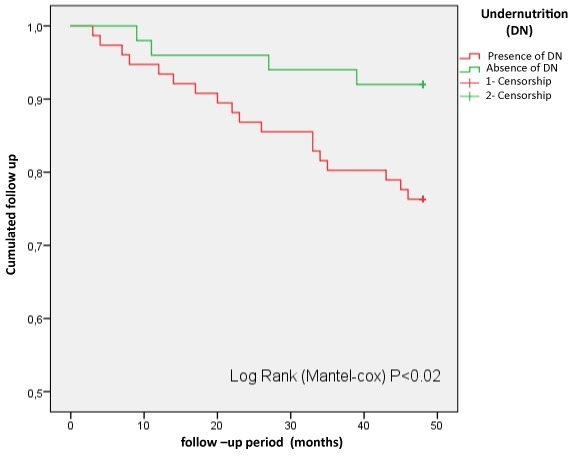
Survival curves of patients according to nutritional status

Protein-energy malnutrition: survival analysis, depending on nutritional status, by the log-rank method, showed a difference between malnourished and non-malnourished patients (p <0.02). From Figure 5, the survival of malnourished patients was 94% at 12 months, 84% at 24 months and 75% at 48 months of follow-up. While, the survival of non-malnourished patients was 93% at 48 months of follow-up.

**Figure 6:**
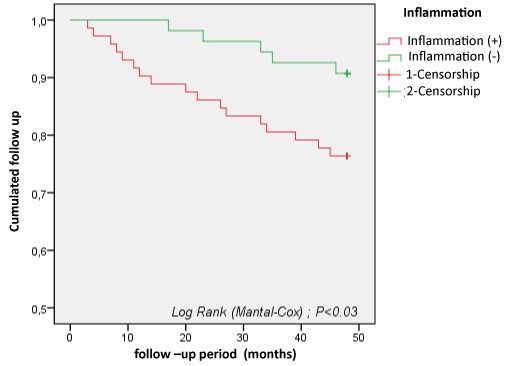
Survival curves of hemodialysis patients according to inflammatory status

Inflammation: the dosage of CRP was performed in 116 patients, 57.75% of whom had a CRP ≥8mg/l. The prevalence of inflammation was 77.27% of deceased patients. The analysis of survival curves showed that the presence of inflammation represents a factor favoring death in our study population (p=0.03). Survival at 4 years was 76% for patients with inflammation and less than 91% for patients without inflammation (Figure 6).

**Figure 7:**
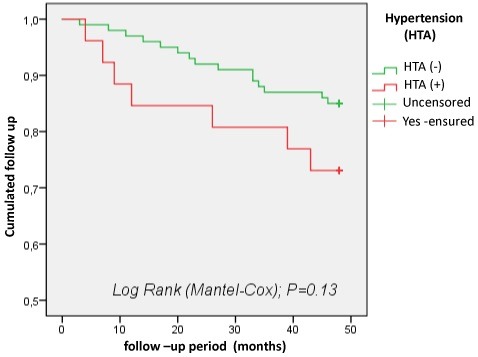
Survival curves as a function of arterial hypertension

Hypertension (HTA): the prevalence of arterial hypertension was 78.44% (65.51% of survivors versus 12.93% of deceased patients). However, this difference was not statistically significant (p=0.15). According to the survival curve, hypertension was not a factor contributing to death in our population (p=0.13). In fact, the 4-year survival was 85% for non-hypertensive patients and 71% for hypertensive (Figure 7).

## Discussion

For many years, the variability in mortality of HD patients between different countries has been widely documented. Our study provides a set of indicators on the survival and causes of death of patients on ESRD treated with hemodialysis. During our study, 22 patients died (17.46%), whose etiology of death was mainly cardiovascular disease (22.72%). Our results also revealed that advanced age, undernutrition or cardiovascular disease, and inflammation are predictive factors for overall mortality of HD patients independently or when associated, thereby decreasing their life expectancy. Worldwide, end-stage chronic kidney disease accounts for about 1% of all deaths. Between 1990 and 2010, the mortality rate from ESRD increased by 15% while the all-cause mortality rate decreased by 21% [11]. The burden of chronic kidney disease on mortality is significant and continues to increase, which requires, even more, optimal medical management of these patients. Several publications have confirmed the excess of cardiovascular morbidity and mortality in patients with renal insufficiency and in particular, those who undergo chronic extra renal treatment. Nearly half of the deaths occurring in dialysis patients have a cardiovascular cause [7, 12].

In our study, they were diagnosed in 22.72% of our patients, this value is probably underestimated, and especially that the cause of death was undetermined for 54.54% of the subjects. This proportion appears to be much lower than many studies [13] and is also higher than what was reported in other previous studies of our team (13.1%) [14]. The prevalence of mortality in HD patients reported by registries is 20 to 35% lower in Europe and Japan compared to the United States [15]. USRDS (United States Renal Data System) patient comparison with those of Registro Lombardo Dialisi E Trapianto (Lombard Registry of Dialysis and Transplantation) shows a relative risk (RR) of mortality of 0.71 (p<0.0001) of Italian patients, after adjustment for different demographic factors and comorbidities [16]. In our work the RR of mortality was 2.91 times higher in the presence of cardiovascular diseases. It is possible that some of the differences observed between countries may be related to the very different prevalence of cardiovascular diseases [17], which is by far the leading cause of death in HD patients. Hypothetically, 10,000 deaths could be avoided in the United States each year if the albumin level was higher than 38g/l in chronic HD patients [18].

However, there are other factors associated with high mortality in HD patients, including malnutrition which is a formidable but not unavoidable complication in chronic hemodialysis and is a major public health problem as evidence of numerous studies [19]. This protein-energy deficit is a determining factor in patients progressive prognosis and has a negative impact on their survival [6, 8, 9, 20]. A French study of patients over 80 years old who started dialysis showed that good nutritional status, early management and good patient autonomy were associated with better survival at 12 months (87% versus 17%) in comparison with a group of patients malnourished and taken care of late [21]. The etiological factors involved are multiple, among which we quote; inappetence-anorexia, inflammatory processes, comorbidities and polymédication [19, 20]. Although abnormal nutritional status is frequently reported in HD patients, unfortunately, there is no “gold standard” and universal marker of undernutrition [22]. However, different markers such as albumin serum, creatinine and Body Mass Index (BMI) have been correlated with increased mortality [23].

One of the first markers studied is albumin with a standard adopted by international companies of 38g/l measured by an immunonephelemetry method [24]. The exploration of the nutritional status, revealed that 77.27% of the deceased patients have an albumin level <38g/l which represents a percentage of 14.64% of our total population. This result is lower than what was found in the Kalantar-Zadehet study and all carried on 58.058 patients (19%) [25]. Many studies have shown that hypoalbuminemia is a good predictor of morbidity and mortality, a 10-year cohort study has indicated a high risk of mortality in HD patients with serum albumin levels below 3.8g/dl [26]. Our results show that malnourished patients have a mortality RR of 1.85 times higher than non-malnourished patients (albumin ≥38g/l) which aligns with data from the literature [27] Friedman and Fadem have shown that serum albumin levels should be used with caution as nutritional markers in patients with chronic kidney disease, because low levels of serum albumin in HD patients are known to be associated with malnutrition and inflammation [28].

It is very likely that much of the influence of nutritional biochemical parameters, particularly albumin, on the morbidity and mortality of dialysis is explained by the relationship between inflammation and these parameters [29]. It has been reported that in the presence of chronic inflammation, malnourished patients have very low albumin levels, a sign of severity of malnutrition or a reflection of resistance to treatment [15]. In our study, we also observed an inflammatory state in 56.87% of patients characterized by serum CRP concentration. The latter was very high among the deceased who were significantly older than the survivors. This is in agreement with the data from previous results of our team [14]. The involvement of inflammatory processes is associated with significant morbidity and mortality, Kaysen *et al.* showed for the first time that CRP was frequently elevated in dialysis patients, with a very strong negative correlation with markers of nutritional status [30]. Other teams have largely confirmed this significant prevalence of chronic inflammation in this population. This chronic inflammatory state is associated with various complications, including increased anemia secondary to CKD and impaired nutritional status [31].

These data are similar to older studies that have shown a greater frequency of cardiovascular damage in cases of hypoalbuminemia [32], illustrating the relationship between chronic inflammation, impaired nutritional status, cardiovascular morbidity and mortality that led to propose the name of syndrome “MIA” for Malnutrition - Inflammation - Artherosclerosis [29]. In addition, the results of the current study showed no significant difference between BMI and survival in patients undergoing hemodialysis (P=0.654). In the overall population, there is a significant association between BMI and mortality [33]. However, a number of clinical studies have demonstrated that in HD patients, BMI was inversely related to mortality [34]. All these results suggest the importance of the management of undernutrition in HD patients. It has been reported that the treatment of malnutrition in HD leads, if successful, to a better quality of life for patients and contributes to the decrease in overall mortality [8]. DOPPS observations (Dialysis Outcomes and Practice Patterns Study) [35] have improved some simple criteria (such as increased blood flow) to increase the survival of dialysis patients in some countries. The same study could show the association between nutrition and various parameters such as inflammation or dialysis dose. However, our study remains limited and investigations need to be conducted on a larger sample to further determine the causes of increased risk of mortality in HD patients.

## Conclusion

An early and repeated evaluation of nutritional status is therefore essential in the follow-up of the dialysis patient. Simple actions in the management of our patients such as optimizing the dialysis dose, biocompatibility of membranes, enhancement of the arteriovenous fistula, the use of different concentrations of dialysis bath potassium and calcium could improve nutritional parameters by decreasing chronic inflammation and thus reducing the risk of mortality.

### What is known about this topic

- Mortality in the chronic renal deficient patient is high compared to the general population;
- Mortality is partly explained by the presence of important co-morbidities;
- Protein-energetic malnutrition of the dialysis patient is frequent.

### What this study adds

- Mortality is high in our cohort;
- Cardiovascular disease, malnutrition and inflammation are predictive factors of mortality;
- Treatment and early management of these factors are essential to reduce mortality in our patients.

## Competing interests

The authors declare no competing interests.
